# Perioperative Ozoralizumab Management for Patients with Rheumatoid Arthritis Who Underwent Orthopaedic Surgery: A Retrospective Case Series

**DOI:** 10.3390/jcm15041422

**Published:** 2026-02-11

**Authors:** Keiichiro Nishida, Yoshihisa Nasu, Ryozo Harada, Ryuichi Nakahara, Masahiro Horita, Masamitsu Natsumeda, Shuichi Naniwa, Toshifumi Ozaki

**Affiliations:** 1 Locomotive Pain Center, Faculty of Medical Development Field, Okayama University, Okayama 700-8558, Japan; p0t304rt@okayama-u.ac.jp (R.N.); masahiro.horita@gmail.com (M.H.); 2 Department of Orthopaedic Surgery, Okayama City Hospital, Okayama 700-8557, Japan; nasu_y@flute.ocn.ne.jp; 3 Department of Orthopaedic Surgery, Kurashiki Sweet Hospital, Okayama 710-0016, Japan; roro_ryo11@hotmail.com; 4 Department of Musculoskeletal Health Promotion, Science of Functional Recovery and Reconstruction, Okayama University Graduate School of Medicine, Dentistry and Pharmaceutical Sciences, Okayama 700-8558, Japan; 5 Department of Orthopaedic Surgery, Faculty of Medical Development Field, Okayama University, Okayama 700-8558, Japan; 6 Rheumatic Disease Center, Mabi Memorial Hospital, Kurashiki 710-1313, Japan; zaraspook3hook@yahoo.co.jp; 7 Department of Orthopaedic Surgery, Faculty of Medicine, Dentistry and Pharmaceutical Sciences, Okayama University, Okayama 700-8558, Japan; me19061@s.okayama-u.ac.jp (S.N.); tozaki@md.okayama-u.ac.jp (T.O.)

**Keywords:** delayed wound healing, discontinuation, ozoralizumab, orthopaedic surgery, perioperative management, rheumatoid arthritis, surgical site infection

## Abstract

**Background/Objectives**: Launched in Japan in 2022, ozoralizumab (OZR) is a novel, anti-tumour necrosis factor (TNF)-α inhibitor for treating rheumatoid arthritis (RA) that is refractory to conventional therapies. However, there is a lack of evidence regarding its perioperative management. **Methods**: This retrospective case series included nine patients with RA who underwent a total of 12 either RA-related (*n* = 9) or unrelated (*n* = 3) orthopaedic procedures. We reviewed patient demographics, surgical procedures, perioperative OZR discontinuation periods, and postoperative complications. **Results:** The mean preoperative OZR discontinuation period was 15.8 days (range, 2–25 days). Sutures were removed at a mean of 12.8 days postoperatively (range, 11–14 days) after adequate wound healing had been confirmed. The mean total discontinuation period was 34.9 days (range, 27–43 days). No cases of surgical site infection (SSI) or delayed wound healing (DWH) were observed during a minimum follow-up period of three months. One patient experienced a disease flare before OZR was restarted. **Conclusions:** Preoperative OZR discontinuation for up to four weeks appeared to be safe in this cohort. These findings may assist orthopaedic surgeons in determining an appropriate perioperative discontinuation strategy for OZR that minimises SSI and DWH risk while reducing the likelihood of RA flare.

## 1. Introduction

Launched in Japan in December 2022, ozoralizumab (OZR) is a novel, anti-tumour necrosis factor (TNF)-α inhibitor for patients with rheumatoid arthritis (RA) who display an inadequate response to conventional therapies [1]. OZR is a small-molecule antibody preparation based on Nanobody^®^ (Ablynx NV, Ghent, Belgium) technology, consisting of a single-domain antibody derived from the variable region of llama heavy-chain antibodies. With a molecular weight of 38 kDa—approximately a quarter that of conventional immunoglobulin G [2]—OZR may access disease target sites that are less accessible to traditional antibodies.

Preclinical studies have demonstrated that OZR has an extended half-life due to its albumin-binding moiety, allowing for its administration at four-week intervals [3]. However, its prolonged retention at inflammatory sites raises concerns regarding perioperative safety in orthopaedic surgery. Although several guidelines address the perioperative management of established TNF inhibitors [4,5,6], no data are currently available for OZR. This study is the first to describe the perioperative clinical course of patients with RA undergoing orthopaedic surgery while receiving OZR.

## 2. Patients and Methods

This retrospective case series was conducted in accordance with the Declaration of Helsinki and approved by the Ethics Committee of Okayama University (No. 2505-043) on 17 October 2025. Written informed consent was obtained from all participants. In this study, data were collected from all patients with RA who underwent orthopaedic surgery at our institutions between 1 September 2020 and 31 March 2025 while receiving OZR therapy. All patients fulfilled the 1987 revised American College of Rheumatology criteria for RA [7].

Nine patients were included (six women and three men), with a mean age of 62.7 years (range, 40–82) and a mean disease duration of 31.9 years (range, 10–49). The mean preoperative DAS28-CRP was 2.4 (range, 1.24–3.95). Four patients used concomitant prednisolone (range, 3.5–10 mg/day), and none had diabetes mellitus. One patient (case No.5) received 80 mg/month of OZR during a clinical trial and underwent surgery after withdrawal. The remaining eight patients received treatment with OZR at 30 mg/month. The patient characteristics before surgery are summarised in Table 1.

Collected data included surgical indications, procedure types, preoperative OZR discontinuation period, time to suture removal, total discontinuation period, and postoperative complications, including surgical site infection (SSI), delayed wound healing (DWH), and RA flare. SSI was defined according to the 2013 United States Center for Disease Control and Prevention guidelines for the prevention of SSI by National Healthcare Safety Network (available at: https://apic.org/Resource_/TinyMceFileManager/Academy/ASC_101_resources/Surveillance_NHSN/ASCA_NHSN_SSI_Surveillance_2013.pdf) (accessed on 13 January 2026). DWH was defined as cases where suture removal was later than 2 weeks after surgery or where re-suturing was required [8].

## 3. Results

The patients underwent a total of 12 procedures related (*n* = 9) or unrelated (*n* = 3) to the RA. One patient (case No.2) underwent two surgeries within a 10-month interval. Another (case No.4) suffered multiple traumas two days after OZR administration; this patient underwent emergency surgery for an open fracture of the femur on the day of injury, and open reduction and internal fixation for a fractured radius and posterior lumbar fusion with vertebroplasty for lumbar fracture 8 days after injury. The mean preoperative OZR discontinuation period was 15.8 (range, 2–25) days. Sutures were removed at a mean of 12.8 (range, 11–14) days postoperatively after wound healing had been confirmed. The mean total OZR discontinuation period was 34.9 (range, 31–37) days. No cases of SSI or DWH were observed during at least three months of follow-up. One patient (case No.4) experienced a flare-up of the disease before restarting OZR (Table 2).

**Figure 1:**
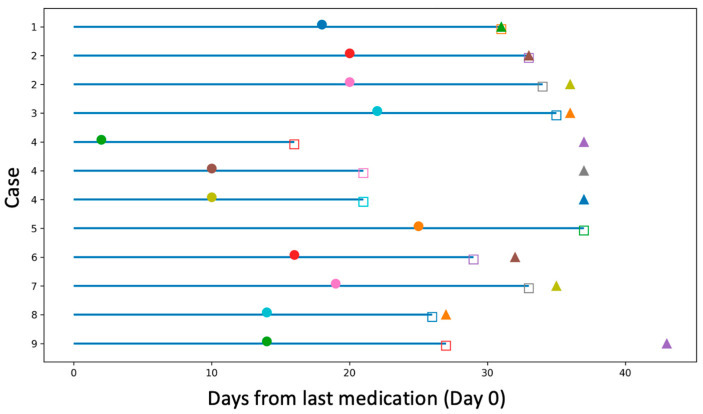
Swimmer plot showing the timing of medication discontinuation, surgery (**●**), suture removal (**□**), and treatment resumption (**▲**) in each case. The horizontal axis represents days from the last medication dose (Day 0). Each horizontal bar corresponds to an individual surgical episode; identical case numbers indicate multiple procedures performed in the same patient. The absence of a triangle indicates cases in which treatment was not resumed during the observation period.

The timing of medication discontinuation, surgery, suture removal, and treatment resumption was visualised using a swimmer plot, with time expressed as days from the last medication dose (Day 0) and repeated procedures in the same patient shown as separate entries (Figure 1).

## 4. Discussion

It has been recognised that continuing biologic disease-modifying anti-rheumatic drugs (bDMARD) in the perioperative period of orthopaedic surgery poses the potential risk of increasing SSI and DWH [9]. Longer withdrawal periods decrease such risks, but increase the chance of disease flare-ups. The patient panel participating in the ACR/AAHKS guidelines for total hip and knee arthroplasties considered the risk of infection to be much more important than the risk of flare [10]. As the British Society of Rheumatology guideline stated, “the potential benefit of preventing post-operative infections by stopping biologics should be balanced against the risk of a peri-operative flare in disease activity” [11]. The Japan College of Rheumatology clinical practice guidelines for managing rheumatoid arthritis recommend discontinuing bDMARD in the perioperative period of orthopaedic surgery (strength of recommendation: weak) [6]. In terms of the bDMARD discontinuation period, several guidelines agree that they should be withheld one dosing cycle before surgery and restarted after good surgical wound healing has been confirmed, usually around 14 days after the surgery [12,13]. However, there are no available data on OZR management, as perioperative experience with this relatively new drug is limited.

This retrospective case series is the first to report the clinical course of RA patients who underwent orthopaedic surgeries under OZR disease control. In this study, four of the nine patients used glucocorticoid (GC), while seven used conventional synthetic disease-modifying anti-rheumatic drugs (csDMARDs), including methotrexate (MTX). GC and csDMARDs may have provided a bridging effect in controlling disease activity during OZR discontinuation. Because it has been suggested that withholding MTX perioperatively increases the risk of flares without increasing the rate of SSI and DWH, most guidelines recommend continuing MTX use throughout this period [13]. In addition, since bDMARDs are typically introduced as add-on therapy when disease control is inadequate, the effect of the continued use of GC and csDMARDs on disease flare is likely limited compared to perioperative bDMARD discontinuation.

Whether surgery timing should be determined according to their half-life or administration interval has not been well addressed, particularly since the latter is decided based on drug pharmacokinetics (PK) [14]. For TNF inhibitors with similar half-lives, such as adalimumab and certolizumab pegol (both of which have half-lives of approximately 14 days) [11], surgery is recommended at 3 weeks after the last dose, i.e., within the period when effective blood concentrations are maintained. It is recommended that another TNF-inhibitor, Golimumab (also with a half-life of approximately 14 days), be withheld for 4 weeks before surgery (after one dosing interval). In our experience, similar perioperative management did not pose a risk for postoperative infection- or wound healing-related complications [15]. It remains unclear why surgery was safe even when plasma concentrations remained within the therapeutic range. The uniquely small molecular weight of OZR may not be relevant to postoperative infection or wound healing.

In this study, all procedures were performed within 4 weeks after OZR discontinuation. Takeuchi et al. investigated the PK profile of OZR in Japanese patients with RA from the OHZORA and NATSUZORA trials. They found the t_1/2_ of OZR to be 18.2 days due to its ability to bind to human serum albumin [16]. The PK simulation showed that the estimated trough concentration of OZR was 1.2 μg/mL when 30 mg was administered at 6-week intervals, which was not below the cutoff value (1.0 μg/mL) derived from the ROC analysis of ACR20 or ACR50 response rates and trough concentrations [16]. Therefore, theoretically, if surgery is performed within 4 weeks (one dosing cycle) of the last OZR administration and OZR is restarted at two weeks postoperatively after the confirmation of wound healing, the plasma OZR concentration would not decrease below therapeutic levels. However, the effective blood concentration cutoff varies per patient and is influenced by body weight, severity of RA-related inflammation, immunogenicity, and concomitant medications such as MTX. It should be noted that pharmacokinetic extrapolation methods have not yet been directly validated in perioperative care.

One of the important observations of the present study was the absence of wound-related complications, even in patients (Case No.4) who sustained multiple traumas and underwent emergency surgery on day 2 after OZR administration. As this patient had additional surgery 8 days later, systemic inflammation persisted, with CRP levels exceeding 70 mg/L until day 14 after traumatic injury. The absence of SSI or DWH, coupled with postoperative disease flare, might be related to the depletion of initial high-concentration OZR due to bleeding from the multiple traumas and surgical injury; alternatively, it might be related to the increased OZR consumption due to elevated TNF production during systemic inflammation. Surgeons should also consider the clinical status of patients, such as their age, disease duration and activity, surgery type, and comorbidities, all of which might influence SSI and DWH development.

There is no clear evidence regarding the optimal time to restart bDMARDs in the postoperative setting, and this approach is based on standard precautions that warn against its use in patients with active infection, such as those with open wounds [10]. In patients with rheumatic diseases undergoing total joint arthroplasty, Goodman et al. described that “antirheumatic therapy should be restarted once the wound shows evidence of healing, any sutures/staples are out, there is no significant swelling, erythema, or drainage, and there is no ongoing nonsurgical site infection, which is typically ~14 days” [10]. In the current study, no complications occurred in the surgical wound, and the average period until stitch removal was approximately 13 days.

Several limitations of this study should be acknowledged. Firstly, its small sample size, absence of a control group, and retrospective nature limit the generalizability of the conclusions regarding OZR’s perioperative safety. In addition, due to the small sample size, we were unable to compare the results of low- and high-risk procedures to determine whether the preoperative discontinuation period of up to 4 weeks is equally safe across different levels of surgical complexity. Secondly, most patients had relatively well-controlled disease activity without major metabolic comorbidities such as diabetes mellitus, which might affect the SSI and DWH rates. Thirdly, only one patient received the higher 80 mg dose of OZR, limiting conclusions regarding dose-dependent effects.

## 5. Conclusions

In conclusion, the present study provides preliminary clinical evidence that withholding OZR up to 4 weeks before orthopaedic surgery and restarting it after wound healing has been confirmed to represent a reasonable perioperative management strategy, although individualised decision-making remains essential. More data from larger prospective or retrospective multicentre studies are needed to establish proper perioperative OZR management.

## Figures and Tables

**Table 1:** Patient backgrounds.

Case No.	Gender	Age (Years)	Disease Duration (Years)	BMI	Dose of Ozoralizumab/Month (mg)	Dose of MTX/Week (mg)	Other csDMARDs	Dose of GC/Day (mg)	Pre-Op. CRP (mg/dL)	Pre-Op. DAS28-CRP
1	F	66	49	19.0	30	0	IGU	3.5	0.35	2.76
2	F	69	48	18.7	30	8	—	0	0.07	1.38
2	F	70	49	18.7	30	8	—	0	0.18	1.24
3	F	40	37	27.3	30	8	—	10	2.4	3.1
4	M	62	15	28.4	30	0	TAC	0	—	—
5	F	40	10	29.5	80	12	—	7	0.34	1.72
6	M	82	23	23.3	30	0	—	0	0.04	2.91
7	M	63	32	19.6	30	0	SASP, IGU	5	0.99	3.95
8	F	72	24	21.2	30	14	IGU	0	0.1	3.23
9	F	62	29	23.1	30	0	—	0	0.1	1.28
Average		62.6	31.6	22.9		5.0		2.6	0.5	2.4

M: male; F: female; IGU: Iguratimod; TAC: Tacrolimus; SASP: Salazosulfapyridine; GC: Glucocorticoid; MTX: Methotrexate.

**Table 2:** Surgery type, perioperative management, and outcomes.

Case No.	Side	Pathology	Procedures	Preoperative Discontinuation Period (Days)	Period Until Removal of Stitches	Total Discontinuation Period (Days)	DWH	SSI	Flare-Up of the Disease
1	R	Foot deformity	Lambrinudi arthrodesis	18	13	31	—	—	—
2	R	Thumb deformity	Thumb reconstruction *	20	13	33	—	—	—
2	R	Finger deformity	Silicon implant arthroplasty **	20	14	36	—	—	—
3	R	EPL rupture	Tendon transfer	22	13	36	—	—	—
4	L	Open fracture of femur	ORIF ***	2	14	37	—	—	+
4	L	Fracture of radius	ORIF ***	10	11	37	—	—	
4	—	L1 fracture	Posterior lumbar fusion, vertebroplasty	10	11	37	—	—	
5	L	Hip joint destruction	Total hip arthroplasty	25	12	not restarted	—	—	—
6	L	Knee joint destruction	Total knee arthroplasty	16	13	32	—	—	—
7	L	Elbow joint destruction	Total elbow arthroplasty	19	14	35	—	—	—
8	L	Elbow joint destruction	Revision total elbow arthroplasty	14	12	27	—	—	—
9	L	Foot deformity	Resection arthroplasty	14	13	43	—	—	—
Average				15.8	12.8	34.9			

* Interphalangeal (IP) joint arthrodesis, metacarpophalangeal (MP) joint silicon implant arthroplasty, carpometacarpal (CM) joint arthroplasty: ** Arthroplasty for 2–5th MP joint, *** ORIF: open reduction and internal fixation, R: right; L: left; SSI: surgical site infection, DWH: delayed wound healing.

## Data Availability

The data in this study are included within the article and available from the corresponding author on reasonable request.

## Abbreviations

The following abbreviations are used in this manuscript:

OZR	Ozoralizumab
TNF	Tumour necrosis factor
DWH	Delayed wound healing
SSI	Surgical site infection
IGU	Iguratimod
TAC	Tacrolimus
SASP	Salazosulfapyridine
GC	Glucocorticoid
MTX	Methotrexate
csDMARD	Conventional synthetic disease-modifying anti-rheumatic drugs
bDMARD	Biologic disease-modifying anti-rheumatic drugs
PK	Pharmacokinetics
ORIF	Open reduction and internal fixation
IP	Interphalangeal
MP	Metacarpophalangeal
CM	Carpometacarpal
